# Supplementary material for: Main drivers of regional value chains in CAFTA: Does trade facilitation matter?
Source: PLoS One. 2023 Dec 7;18(12):e0289775. doi: 10.1371/journal.pone.0289775 (PMC10703318; doi:10.1371/journal.pone.0289775)
Supplement: Attachment — Submitted filename: renamed_2ac79.docx [file pone.0289775.s001.docx]

**Response to reviewers**

Dear editors and reviewers,

Thank you very much for these detailed and useful feedbacks on our paper. We have considered your comments and have incorporated almost all your suggestions in the revised version of our paper. We also have a detailed response to each comment in the below. In the paper, the changes made are highlighted in yellow. We feel that by revising our paper according to your constructive suggestions, the paper has been greatly improved.

**Comments from the editors and reviewers:**
- **Referee 1**-

The authors worked on interesting research topic "Main drivers of regional value chain in CAFTA: Does trade facilitation matters". The article is acceptable after some minor revisions.

First, the authors used many abbreviations - especially in abstract - some of them needs to be explained right from start of the article.

Response: Thanks a lot for your valuable comments. We have supplemented our previous oversight by explaining abbreviations as China and ASEAN Free Trade Area (CAFTA), domestic value added (DVA), returned added value (RDV), and domestic value added reexported to third country (DVA INTrex) in the abstract.

Further, the article lacks the discussion. The section 5 of the article is "Conclusions and Discussion". In the subsections no discussion is found. The authors should discuss the results before the conclusion by adding separate section "discussion" which should be sufficient enough according to the size of the results.

Response: Thanks for your valuable comments. According to your and the second reviewer’s suggestion, we tried to add the discussion part of the results. We have added a discussion section-*Section 4.3*, in which we discussed the results and compared them with relevant previous literature. For example, we compared the research results of trade facilitation on DVA-D, DVA-INTrex, and RDV with previous literature (Line 576-589), and found out that the results showed consistency with previous literature. Moreover, we pointed out that this study further revealed that in regions like CAFTA where tariffs and trade barriers between internal countries are relatively low, the key factor affecting the value of DVA-INTrex and RDV is the business environment(*bus*). Please see the revised part in *Section 4.3* (Line 590-598).

- **Referee 2**-

To further improve the quality of the manuscript, the following suggestions are proposed:

1. In the abstract, also add that for what the abbreviation DVA stands

Response: Thanks a lot for your valuable comments. We have supplemented our previous oversight by explaining abbreviations as China and ASEAN Free Trade Area (CAFTA), domestic value added (DVA), returned added value (RDV), and domestic value added reexported to third country (DVA INTrex) in the abstract.

2. No doubt, the presentation of a diagrammatic conceptual framework is an elaborative contribution of the study. But it would increase the understand about the trade facilitating variables, if each of the different sections (A, B, C, D, and E) of the diagram (Fig.2) are a bit explained further.

Response: Thanks for your positive and valuable comments. We are very sorry for the confusing signs, as sections (A, B, C, D, and E) of the diagram (Fig.2) were not in a one-to-one correspondence to the explanation below. In the revised part, we have made it clear that the ABCDE in Figure 2 corresponds to market access& targets and trade barriers (mar), customers efficiency (cus), information and communication technology (ict), infrastructure construction (inf), and business environment (bus), respectively. We provided detailed explanations in Line 165-167. Furthermore, we also explained their specific connotations in Line 165, 173, 191, 208, 222 and 237.

3. There is need to add the theoretical underpinnings of the study as well, for example different models of trade, pertinent to this study, like, the gravity theory of international trade etc.

Response: Thanks for your suggestions. In the revised part, we completed previous research in the academic community on the gravitational model of international trade before using Quadratic Assignment analysis. And we also explained why QAP analysis method was chosen in this article. (Line 324-339)

4. Fig.6 (depicting SNA results) is fade and does not clearly exhibit the trade related networking among the CAFTA countries. Similarly, there is no explanation about the figure in terms of what the figure is showing through dark and dim multicolored lines in different time periods.

Response: Thanks for valuable comments. We’re sorry that the description of Figure 6 was unclear and lacked depth. In the revised part, we provided a detailed description of the flow of trade value added between countries and its profound significance in regional value chains and trade products in 2001, 2010, and 2016. And we would like to explain that, Fig.6a to 6i represent the three types of trade added value at three time nodes. Therefore, Fig.adg use the same legend, as well as Fig.beh and Fig.cfi, in order to make it easier to compare the changes in the value added of trade in different years.

5. Table 8 claims to exhibit QAP results of different TFI pillars’ impacts on three value-added in trade networks, whereas only the finding of two trade networks (DVA-INTrex and RDV networks) are there.

Response: Thank you for pointing out this error. We have adjusted the name of Table 8 to QAP results of different TFI pillars' impacts on DVA-INTrex & RDV trade networks. (Line 573)

6. QAP analyses (QAP correlations and QAP regressions for the years 2010 and 2016) are done to investigate the influence of the trade facilitation factors on the intermediate goods trade. Those trade facilitation factors or pillars are: domestic business environment(bus), domestic and foreign market access (mar), customs efficiency (cus), availability and quality of transport infrastructure and services (inf), and information and communication technology (ict). But in the QAP regressions (as evident from the findings given in Table 8), why all the five different pillars of trade facilitation are not included.

Response: Thank you for your suggestion. All the five different pillars of trade facilitation have been included in the QAP regression. In fact, the regression results showed that the domestic business environment (*bus*) had a significant impact on indirect and transferred value-added in 2010 and 2016. And customers efficiency (*cus*) and information and communication technology (*ict*) were significant in 2016. Availability and quality of transport infrastructure and services (*inf*) and domestic and foreign market access (*mar*) were not significant. The relevant results correspond to *sum-bus, sum-mar, sum-cus, sum-inf*, and *sum-ict*, respectively. And we explained the reason for not being significant in Line 570-572.

7. The findings of the study are discussed least in comparison with the relevant previous literature

Response: Thanks for your valuable comments. According to your and the first reviewer’s suggestion, we tried to add the discussion part of the results. We have added a discussion section-*Section 4.3*, in which we discussed the results and compared them with relevant previous literature. For example, we compared the research results of trade facilitation on DVA-D, DVA-INTrex, and RDV with previous literature (Line 576-589), and found out that the results showed consistency with previous literature. Moreover, we pointed out that this study further revealed that in regions like CAFTA where tariffs and trade barriers between internal countries are relatively low, the key factor affecting the value of DVA-INTrex and RDV is the business environment. Please see the revised part in *Section 4.3* (Line 590-598).

8. Policy implications (section 5.3 of the manuscript) could not be so specific, like stated in the lines 574 and 575: “promote e-commerce and paperless reform, strengthen the individual's Internet skill, eliminate high cost transactions and serious time delays, and reduce administrative corruption”.

Response: Thanks for your valuable comments. We have removed the original inappropriate statements and rewritten the policy implications section in Line 644-651.
